# The Effect of Vocabulary Depth and Breadth on English Listening Comprehension Can Depend on How Comprehension Is Measured

**DOI:** 10.3389/fpsyg.2021.657573

**Published:** 2021-05-25

**Authors:** Yuzhi Luo, Hongwen Song, Li Wan, Xiaochu Zhang

**Affiliations:** ^1^School of Foreign Languages, Anhui Agricultural University, Hefei, China; ^2^School of Humanities and Social Science, University of Science and Technology of China, Hefei, China; ^3^Chinese Academy of Sciences Key Laboratory of Brain Function and Disease, School of Life Sciences, University of Science and Technology of China, Hefei, China; ^4^Hefei Medical Research Center on Alcohol Addiction, Anhui Mental Health Center, Hefei, China; ^5^Academy of Psychology and Behavior, Tianjin Normal University, Tianjin, China; ^6^Division of Life Science and Medicine, Department of Radiology, The First Affiliated Hospital of USTC, Hefei National Laboratory for Physical Sciences at the Microscale and School of Life Science, University of Science and Technology of China, Hefei, China; ^7^Centers for Biomedical Engineering, School of Information Science and Technology, University of Science and Technology of China, Hefei, China

**Keywords:** vocabulary depth, vocabulary breadth, listening comprehension, language test, language leaning

## Abstract

This study examines the relative contribution of vocabulary breadth (VB) and vocabulary depth (VD) to three different listening comprehension measures. One hundred and thirteen English majors were given VB and VD tests, and three listening comprehension tests. Based on three pairs of hierarchical multiple regression analyses, we found that the relative contribution of VB and VD varied across the three listening comprehension tests. Specifically, for the listening test with an expository text dictation to assess integrative skills, both VB and VD made a unique positive contribution to comprehension, but this was greater in the case of depth. For the listening test involving narrative conversations to assess literal comprehension, neither VB nor VD (after controlling for each other) could independently predict comprehension, whereas for the listening test that comprises expository passages to assess inferential comprehension, VD could separately predict comprehension but VB could not. These findings suggest that the relative contribution of VD and VB to listening comprehension may depend on how a listening test is constructed. Therefore, the findings will contribute to listening comprehension and vocabulary knowledge research, and vocabulary teaching and learning.

## Introduction

According to the lexical quality hypothesis (Perfetti, 2007), comprehension depends on high-quality lexical representations, and it can be inferred that vocabulary knowledge should play a significant role in second language listening. Many empirical studies have found that vocabulary knowledge relates closely to second language (L2) listening comprehension (Kelly, 1991; Bonk, 2000; Mecartty, 2000; Stæhr, 2009; Matthews and Cheng, 2015; Dabbagh, 2016; Vafaee and Suzuki, 2020; Wallace, 2020). However, there is no consensus on the relative strength of vocabulary depth (VD) and vocabulary breadth (VB) in second or foreign language listening comprehension. Stæhr (2009) found that VB is the basic component of vocabulary knowledge in L2 listening comprehension and that VD cannot predict listening separately. Dabbagh (2016) concluded that only VD can significantly predict the listening comprehension of L2 learners, and that VB does not have such predictive power. To gain a clear picture of the relative effects of the two dimensions of vocabulary knowledge on L2 listening, more studies using multiple measures of listening are required.

This study aims to investigate whether the relative strength of VB and depth in L2 listening depends on how comprehension is constructed. In addition, the relationship between VB and VD depth for intermediate Chinese English learners is explored.

## Literature Review

### Two Dimensions of Vocabulary Knowledge

Vocabulary knowledge is a critical part of second language acquisition (Schmitt, 2010, 2014). Although there is no consensus on the multidimensional nature of vocabulary knowledge, the basic distinction between the breadth and the depth of vocabulary is widely accepted in the field (Read, 1993; Wesche and Paribakht, 1996; Qian, 1999, 2002). VB, the size of vocabulary of a learner, refers to the number of words for which the learner has at least some of the significant aspects of meaning knowledge. Meara (1996) pointed out that vocabulary size is the basic dimension of the lexical competence of learners and argued that learners with a wider vocabulary size are more proficient language users than learners with a limited vocabulary size. Many research results have emphasized that VB is a determining factor for L2 reading and listening comprehension. For example, to obtain an adequate comprehension of a written text, learners need to master a vocabulary size of 8,000–9,000 words, whereas to have good comprehension of a spoken text, learners need to be equipped with a vocabulary size of 6,000–7,000 words (Nation, 2006).

Compared with VB, VD is “a rather loose construct that can be conceptualized in a variety of ways” (Schmitt, 2014, p. 9). Qian (1999) and Read (2004) have proposed that VD might include components, such as pronunciation, spelling, meaning, register, frequency, and morphological, syntactic, and collocation properties (Qian, 1999; Read, 2004). Their conceptualization of VD has some similarities to the term “high-quality lexical knowledge” proposed by Perfetti (2007). The vocabulary of learners includes words of widely varying lexical quality. Words of high quality have bonded phonology, orthography, grammar, and meaning, while words of low quality have missing information or incomplete bonds (Perfetti, 2007). This approach to the construct of VD provides an extensive view of vocabulary knowledge.

A second approach to conceptualizing VD relates to the degree in which words are integrated into the mental lexicon of a learner and reflects the ability of a leaner to associate the word to other related words (Read, 1988, 1993; Meara, 1996; Wilks and Meara, 2002). Under this framework, a widely adopted measure used to investigate VD is the Word Associates Test of Read (Read, 1988, 1993). The test is intended to measure both semantic and collocational associations through a receptive word association task in a practical fashion.

Another important approach to conceptualizing VD relates to receptive and productive vocabulary knowledge (Schmitt, 2014). It is broadly acknowledged that there is a distinction between receptive and productive vocabulary knowledge (Fan, 2000; Nation, 2001; Webb, 2005, 2008). Receptive mastery of words means that learners are able to comprehend the basic meaning of words, while productive mastery entails that learners are able to recall the forms and usages of words. For receptive purposes, knowing the form-meaning link is enough for a learner, while for productive uses, on top of the meaning, he/she must know all of the word knowledge to produce the appropriate word in the context given. In this sense, VB can be conceptualized as receptive vocabulary knowledge, and VD can be conceptualized as productive vocabulary knowledge (Zhang, 2011; Wang, 2015). Following on from this conceptualization, a widely adopted measure used to assess VB is the Vocabulary Levels Test (Schmitt et al., 2001), and a widely adopted measure used to assess VD is the Productive Vocabulary Levels Test (Laufer and Nation, 1999). The study adhered to this conceptualization of VB and VD and operationalized VB and VD as the scores on the Vocabulary Levels Test and the Productive Vocabulary Levels Test, respectively.

### The Relationship Between Vocabulary Breadth and Vocabulary Depth

Many scholars have studied the relationship between VB and VD. On one hand, some researchers have argued that there is no conceptual distinction between VB and VD because empirical evidence shows a high correlation between them. For example, Vermeer (2001) suggested that VB is indistinguishable from VD because she found high correlations of 0.85 and 0.76 between VB and VD in Dutch monolingual kindergartners and Dutch bilingual kindergartners, respectively. On the other, other researchers have claimed that VB and VD are two different entities, because regression analyses usually illustrate that VD has unique predictive power in addition to VB. For example, Qian (2002) suggested that VB and VD “tap different aspects of vocabulary knowledge” (p. 531). In his study, although a strong correlation of 0.7 between VB and VD was found in University students from different countries, regression analyses indicated that VD added unique predictive power compared to VB alone. Based on a comprehensive review of a large number of empirical studies on the relationship between VB and VD, Schmitt (2014) proposed that the correlation between VB and VD depends on how these two vocabulary dimensions are conceptualized and measured. Additionally, the relationship depends on a variety of factors including the vocabulary size, target words frequency level, and first language of learners. Specifically, “for higher frequency words, and for learners with smaller vocabulary sizes, there is often little difference between size and a variety of depth measures.” However, “for lower frequency words and for larger vocabulary sizes, there is often a gap between size and depth, as depth measures lag behind the measures of size” (Schmitt, 2014, p. 941). Given the complex relationship between VB and VD, more research studies with different measures and different participants are needed to confirm and assess these results.

### The Relationship Between Vocabulary Knowledge and Listening Comprehension

It is generally acknowledged that listening comprehension is an inferential and active cognitive process in which a listener constructs meaning by drawing upon two major knowledge bases: linguistic (including phonological, lexical, syntactic, semantic, or pragmatic knowledge) and non-linguistic (including knowledge of a context or topic, or general knowledge of the world) (Buck, 2001; Rost, 2002; Vandergrift, 2007). To construct the meaning of a spoken input, listeners draw on both knowledge bases through top-down and bottom-up processes. It is assumed that successful listening comprehension is the result of a complex interaction between top-level and bottom-level cues. According to Bonk (2000), to make use of top-level cues and construct an adequate meaning representation of a text, listeners need to recognize a number of words in the input through bottom-level processing. Thus, word segmentation and recognition form the basis of listening comprehension (Rost, 2002). On top of word recognition, many other factors affect L2 listening comprehension, including text type (Shohamy and Inbar, 1991), topic familiarity and background knowledge (Schmidt-Rinehart, 1994), purpose of listening [e.g., whether learners listen for local or global information in the input (Shohamy and Inbar, 1991), and skills measured (Wallace and Lee, 2020)].

Two theoretical models concerning the relationship between vocabulary knowledge and reading comprehension are the instrumentalist hypothesis and the lexical quality hypothesis. According to the instrumentalist hypothesis, vocabulary is the building block of a language. In order to comprehend a text, learners need to know the meaning of words in the text (Anderson and Freebody, 1981). The more words learners know, the better they would be at comprehension. The lexical quality hypothesis (Perfetti, 2007) offers a sound basis for the instrumentalist hypothesis. It speaks of the importance of high-quality lexical representations in L2 reading comprehension (Perfetti, 2007). “A lexical representation has high quality to the extent that it has a fully specified orthographic representation (a spelling) and redundant phonological representations (one from spoken language and one recoverable from orthographic-to-phonological mappings)” (Perfetti and Hart, 2001, p. 68; Perfetti and Hart, 2002, p. 190). In support of the two theories, many empirical studies have found significant correlations between vocabulary knowledge and L2 reading comprehension (Laufer, 1992; Qian, 1999, 2002; Hu and Nation, 2000; Mecartty, 2000; Henriksen et al., 2004; Stæhr, 2008; Ma and Lin, 2015; Makhoul and Sabah, 2019).

The instrumentalist hypothesis and the lexical quality hypothesis can be applied to L2 listening comprehension. Similarly, in support of the two theories, many empirical studies have found significant correlations between VB and L2 listening comprehension (Kelly, 1991; Bonk, 2000; Mecartty, 2000; Stæhr, 2008). For example, Kelly (1991) analyzed listening errors made by advanced English as a Foreign Language (EFL) learners when transcribing passages from BBC radio news recordings. He concluded that lack of vocabulary knowledge is the main obstacle to successful listening comprehension in advanced L2 learners. With 59 Japanese University students of low-intermediate to advanced English ability as participants, Bonk (2000) investigated the relationship between lexical knowledge and L2 listening comprehension and found that efficient listening strategies may make comprehension of lexically complex texts possible and that most learners seem to need very high lexical familiarity for good comprehension. Mecartty (2000) found that vocabulary knowledge emerged as a significant predictor of listening comprehension, which can account for 14% of listening ability. In sum, all these research results confirm that vocabulary plays an important role in L2 listening comprehension.

It is worth noting that some studies that explored the correlation between VB and L2 listening comprehension have indicated that phonological vocabulary has a stronger correlation with L2 listening comprehension than orthographical vocabulary. For example, Milton et al. (2010) investigated the relationship between vocabulary size score and International English Language Testing System (IELTS) subskills (listening, speaking, reading, and writing) with 30 EFL students. Tests for orthographic vocabulary size (the X-Lex) and phonological vocabulary size (the A-Lex test) were used. In terms of listening, they found that phonological vocabulary (aural vocabulary) displays stronger correlation with listening (*r* = 0.67, *p* < 0.01) than orthographic vocabulary (written vocabulary) (*r* = 0.48, *p* < 0.01). These findings indicate the importance of assessing listening vocabulary through a phonological vocabulary test. However, it is common to assess listening vocabulary knowledge for L2 reading and to assess orthographic vocabulary knowledge for L2 listening in the field. Currently, the effect of a mismatch in modality between a vocabulary knowledge measure and an L2 comprehension task is not completely clear (Zhang and Zhang, 2020).

Nowadays, there is a growing concern over the relative contribution of VB and VD to L2 listening comprehension (Stæhr, 2009; Wang, 2015; Dabbagh, 2016; Vafaee and Suzuki, 2020). There are mainly two kinds of findings. First, VB has a higher correlation with and greater contribution to L2 listening comprehension than VD. For example, with advanced Danish learners of EFL, Stæhr (2009) suggested that VB might be a major contributing factor to successful listening comprehension and that VD did not play a separate role. VB alone accounted for a significant 49% of the variance in listening comprehension, while VD added 2% to the variance already explained by VB. Second, VD has a higher correlation with and greater contribution to L2 listening comprehension than VB. For example, by examining the relationship between VD, VB, and listening comprehension in Chinese students at different levels, Wang (2015) concluded that both VD and VB influenced listening scores significantly, and that the overall effect of VD was significantly greater than that of VB. In addition, after examining the predictive roles of VD and VB in the English listening comprehension of 73 EFL learners, Dabbagh (2016) revealed that VD explained 72% of L2 listening variance and that VB did not make a statistically significant contribution to L2 listening variance. These results indicate that the relative contribution of VB and VD to L2 listening varies across different studies, and that the extent to which VB and VD contribute to L2 listening comprehension is far from clear. More studies are needed to explore the relative strength of contributions of the two dimensions of vocabulary knowledge to L2 listening.

By analyzing the listening task types, we found that the above-mentioned three listening tests across three studies were different. The study of Stæhr (2009) utilized the listening part of the Cambridge Certificate of Proficiency in English (CPE), which includes task types such as multiple choice, sentence completion, and three-way matching. The study of Wang drew upon the listening section of CET-4 (College English Test Band 4) with task types that include multiple choices and sentence completion. The study of Dabbagh used the subsection of the IELTS listening part with task types that include sentence/note/table completion, short answer questions, multiple choices, and diagram labeling.

Specifically, the listening test of Stæhr included 13 multiple choice questions (most of which assessed the inferential understanding of opinions and attitudes of test-takers) (CPE Handbook, 2002). The listening test of Wang consisted of 25 multiple choice questions (about 15 assessed literal understanding while 10 assessed inferential understanding). The listening test of Dabbagh included five multiple choice questions (most of which assessed inferential understanding). Different results might be attributed to different listening comprehension measures in the three studies, that is to say the relative contribution of VD and VB to L2 listening comprehension might vary across different listening comprehension measures.

### The Relative Contribution of Vocabulary Breadth and Depth to Different Second Language Reading Measures

It is worth noting that two studies have revealed that the relative contribution of VB and VD depth to L2 reading varies across different reading measures. In a study of Chinese high school English immersion students, Li and Kirby (2015) found that the relative contribution of VB and VD to reading comprehension depended on how reading comprehension was assessed. Specifically, VB significantly predicted a multiple choice reading comprehension task, which required a general understanding of the text, while VD contributed to summary writing, which required a deeper text processing. Further, Zhang and Yang (2016) suggested that the extent to which VB and VD were relatively contributive to reading comprehension varied according to reading texts and tasks. Specifically, VB was a more important contributor for reading tasks to test literal understanding, while VD was a more significant contributor for reading tasks to test inferential comprehension. Taken together, these findings indicate that the relative contribution of VB and VD to L2 reading comprehension varies across different comprehension texts and tasks.

According to Cutting and Scarborough (2006), different reading comprehension measures tap different cognitive processes. Some reading comprehension measures, for example, with multiple choice questions to assess literal understanding may access mainly lower-level skills; but others, for example, with multiple choice questions to assess inferential understanding may demand higher-level skills. Empirical studies have found considerable degrees of similarity between reading comprehension and listening comprehension (Buck, 1992; Petersen et al., 2020). Therefore, it can be inferred that different listening comprehension measures tap different cognitive processes, too. Most importantly, in addition to individual differences in listener characteristics (e.g., vocabulary), performance on L2 listening is also influenced by characteristics of the listening measure (e.g., skills measured) (Wallace and Lee, 2020). Consequently, VB and VD might be expected to contribute differently to different types of comprehension. However, to our knowledge, there has been no research on the relative contribution of VB and VD to different L2 listening comprehension measures.

### The Present Study

Currently, fewer studies have been conducted on the relationship between vocabulary knowledge and L2 listening in comparison with L2 reading. Importantly, the above-mentioned two studies have probed whether the relative contribution of VB and VD to reading comprehension varies across different comprehension texts and tasks. However, to our knowledge, the question of how the relative contribution of VB and VD to L2 listening comprehension performance varies across assessment tasks has received little attention. Based on research designs from the above-mentioned reading studies, this study focused on the relative contribution of VB and VD to three different listening comprehension measures. Specifically, the research questions addressed in the present study were:

1. For L2 learners, is there a significant relationship between VD and VB?
2. For L2 learners, does the relative contribution of VD and VB to listening comprehension depend on how comprehension is assessed?

## Materials and Methods

### Participants

Participants in the first group were 113 second-year English majors (16 males and 97 females) from a Chinese University. Their native language was Chinese. The average age was 20.51± 0.53 years old, and the average years of learning English was 10.34 ± 1.96 years. Almost all the participants had similar educational background, and they learned English in classroom settings. In addition, none of them had experienced living in an English-speaking environment, and neither of their parents were English speakers. The average score in the English proficiency test on their Chinese college entrance examinations was 123.48 ± 7.38 points (the possible maximum score is 150 points). Additionally, the average score in the Test for English Majors-band 4 (TEM-4) was 66.66 ± 9.24 points (the possible maximum score is 100 points). TEM-4 is a national test used to measure the English proficiency of Chinese English majors.

In order to get the discrimination validity of the Vocabulary Levels Test and the Productive Vocabulary Levels Test, a second group of participants (*n* = 120) was recruited. The participants were freshmen majoring in English.

### Instruments

#### The Vocabulary Levels Test

The Vocabulary Levels Test (Version 2) (Schmitt et al., 2001) was used to assess the breadth of vocabulary knowledge. It is composed of five separate sections that include four levels of word frequency (2,000, 3,000, 5,000, and 10,000 vocabulary levels) and an academic vocabulary level. Each level contains 60-word and 30-word explanations displayed in groups of six words and three word explanations (Table 1). Participants are asked to match the words with the given explanations for each group. Each correct answer is given a point. The maximum possible score is 150 points.

**Table 1:** Example items in the Vocabulary Levels Test.

**Word**	**Meaning**
**Example question:**	
1. Business	
2. Clock	Part of a house
3. Horse	Animal with four legs
4. Pencil	Something used for writing
5. Shoe	
6. Wall	
**Example answer:**	
1. Business	
2. Clock	6 Part of a house
3. Horse	3 Animal with four legs
4. Pencil	4 Something used for writing
5. Shoe	
6. Wall	

The test was originally developed by Nation (1983) as a diagnostic vocabulary test for teachers. Based on the older versions of the Vocabulary Levels Test, Schmitt et al. (2001) constructed two new versions, 1 and 2, and explored the reliability of the two versions. The reliability indices (Cronbach's alpha) for different levels of Version 2 are the following: 2,000 level −0.922; 3,000 level −0.927; 5,000 level −0.927; 10,000 level −0.924; and academic −0.960. These indices are consistent with the 0.94 and 0.91 figures explored by Read (1988) for the original Vocabulary Levels Test, indicating that Version 2 provides good reliability. Further, a range of analysis techniques was used to present validity evidence. First, item analysis was carried out and item facility values were the following: 2,000 level −0.783; 3,000 level −0.664; 5,000 level −0.579; 10,000 level −0.290; and academic −0.756. Second, scalability analysis indicated that four frequency sections (2,000, 3,000, 5,000, and 10,000) had a very high degree of scalability (0.978). Third, personal interviews showed that “examinees accept the test and that answers on the test do reflect underlying lexical knowledge” (Schmitt et al., 2001, p. 79). These empirical evidence indicates that the test can provide a valid estimate of the vocabulary knowledge of learners at different frequency levels. Additionally, in this study, an independent samples *T*-test was carried out between the Vocabulary Levels Test scores of the freshmen and those of the sophomores. The *T*-test results showed a significant difference between the two groups (the discrimination validity for the Vocabulary Levels Test was *t* = 8.325, *p* = 0). This indicates that the test has good discrimination validity. Since 2001, the Vocabulary Levels Test (Version 2) has been widely used in vocabulary assessment and vocabulary research studies (Stæhr, 2009; Akbarian, 2010; Zhang, 2011, 2012; Ma and Lin, 2015; Wang, 2015; Zhang and Lu, 2015).

#### The Productive Vocabulary Levels Test

The Productive Vocabulary Levels Test (Version A) (Laufer and Nation, 1999) was employed to measure VD. Modeled on the Vocabulary Levels Test (Nation, 1990), it focuses on a controlled production measure of vocabulary that consists of items from four frequency levels (the 2,000-, 3,000-, 5,000-, and 10,000-word levels) and an academic vocabulary level.

The test samples 18 items in each of the four frequency levels and uses a completion item type. For each item, a meaningful sentence context is provided, and the first letters of a target item are given. Participants are asked to complete a word with the correct form (Table 2). Each correct answer is given a point. The maximum possible score is 90 points.

**Table 2:** Example items in the Productive Vocabulary Levels Test.

Example question: He was riding a bic—.
Example answer: He was riding a bicycle.

Laufer and Nation (1999) conducted a study to check the reliability of the measure. The reliability indices (Kuder–Richardson reliability coefficients KR21) for different levels of Version A are the following: 2,000 level −0.77; 3,000 level −0.81; 5,000 level −0.84; 10,000 level −0.90; and academic −0.84. The results showed that the Productive Vocabulary Levels Test (Version A) is “a reliable, valid, and practical measure of vocabulary growth” (Laufer and Nation, 1999, p. 44). In addition, the Productive Vocabulary Levels Test (Version 2) was widely adopted by some Chinese scholars to assess VD (Zhang, 2011; Wang, 2015). In addition, in this study, an Independent Samples *T*-test was carried out between the Productive Vocabulary Levels Test scores of the freshmen and those of the sophomores. The *T*-test results showed a significant difference between the two groups (the discrimination validity for the Productive Vocabulary Levels Test was *t* = 5.534, *p* = 0). This indicates that the test has good discrimination validity.

#### Listening Comprehension Measures

In this study, three different listening comprehension measures were used. The first listening test was a passage dictation measure. Passage dictation requires students to transcribe the whole passage word for word to measure the listening comprehension ability and proficiency of students in spelling and punctuation. Joynes (1900) argued that the value of dictation “includes not spelling only…but all that belongs to grammar, phrase, or sentence…all that is possible in composition or retranslation (p. 25). Oller (1979) claimed that dictation in which participants need to divide up the stream of speech and to write down what is heard required participants understand the meaning of the material, i.e., relating linguistic context to the extra-linguistic context. The dictation passage (~150 words in length) was read four times. During the first reading, which is read at about a speed of 120 words per minute, students are required to listen and try to understand the meaning. For the second and third reading, the passage is read sentence by sentence or phrase by phrase, with intervals of 15–20 s, and test-takers write down what they have heard. The last reading is read at about a speed of 120 words per minute again; and during this time, test-takers check what they have written. After listening to the dictation passage four times, test-takers are given two min to check their work. This measure takes up ~15 min. The dictation passage is expository. Two raters scored the dictation, and the inter-rater reliability was 0.93. All disagreements were resolved through discussion. The dictation is scored segment by segment. A correct segment is scored a point, and the maximum possible score is 15 points. Mistakes are classified into major and minor mistakes. Major mistakes include word-missing, word-adding, word-changing and tense mistakes, etc. Minor mistakes include slightly misspelled words (1–2 letters misspelled), punctuation, articles, and singular/plural forms. Each major mistake will result in a deduction of 1/2 point, whereas each minor mistake will result in a deduction of 1/4 point. Repetitive mistakes are deducted once. In addition, the maximum deduction for each segment is one point.

The second measure was multiple choice. The participants were asked to listen to three 200-word conversations, each followed by three or four multiple choice questions. In total, there were 10 multiple choice questions, of which 9 were literal (factual) and 1 was inferential. Each conversation was read only once at a speed of 120 words per minute. The participants were asked to make the right choice based on what they had heard. The maximum possible score is 10 points. There are four options (three distracters and a correct answer) for each multiple choice question. Learners cannot get the correct answer just by guessing.

The third measure was also multiple choice. This measure comprises three 200-word expository texts, with each followed by three or four multiple choice questions. There were 10 multiple choice questions in total, of which eight were inferential and two were literal (factual). Each passage was read only once at a speed of 120 words per minute. The participants were asked to make the right choice based on what they had heard. The maximum possible score is 10 points. There are four options (three distracters) for each multiple choice question. Learners cannot get the correct answer just by guessing.

These three listening measures were deliberately chosen for this study. The texts in the dictation and passage comprehension sections were expository. In this way, the effects of text genre on listening comprehension were controlled. In addition, both passage comprehension and conversation comprehension were multiple choice. Thus, the test format effects on listening comprehension could be controlled.

According to the self-report of the participants, none of them had attempted these measures before. The three listening tests are described in detail in Table 3.

**Table 3:** The description of three listening tests.

**Testing contents**	**Task types**	**Skills tested**	**Topics**	**Score**
Dictation	Passage dictation	General understanding, integrative skills	Choosing a career	15
Conversations	Multiple choice	Literal understanding	1. A project team meeting. 2. The pros and cons of advertisements. 3. The hospital rules.	10
Passages	Multiple choice	Information reorganizing inferential understanding	1. A hotel for an international conference. 2. The new museum of industrial and rural life. 3. Safety in dormitory and personal security.	10

Five native speakers experienced in teaching English as a second/foreign language rated the passage difficulty and the topic difficulty of the three listening tests for Chinese intermediate English learners, with 1 indicating that the passage is very simple and 7 that it is very difficult. The dictation passage received a rating of 3.2 (range 2–5) for passage difficulty and 3 (range 2–5) for topic difficulty. The three conversation passages received an average rating of 3 (range 2–4) for passage difficulty and 2.8 (range 2–4) for topic difficulty. The three passages received an average rating of 3.4 (range 2–5) for passage difficulty and 3 (range 2–5) for topic difficulty.

Additionally, frequency levels of words in the three listening tests were analyzed using *Vocabprofile* on the *Compleat Lexical Tutor* website (Cobb, 2021) against frequency-ordered word lists extracted from the British National Corpus (BNC). Over 99% of the words in the dictation test, over 97% of the dialogues test, and over 94% of the passages test were within the 5,000-word frequency range (Table 4).

**Table 4:** Frequency analysis of the lexical content of the three listening tests.

**Frequency level**	**Word families (%)**			**Tokens (%)**			**Cumulative tokens (%)**		
	**Dictation**	**Dialogue**	**Passage**	**Dictation**	**Dialogue**	**Passage**	**Dictation**	**Dialogue**	**Passage**
1,000	72 (83.7)	194 (87)	191 (78.3)	137 (90.1)	672 (92.7)	533 (82.5)	90.1	92.7	82.5
2,000	7 (8.1)	15 (6.7)	30 (12.3)	8 (5.3)	21 (2.9)	40 (6.2)	95.4	95.6	88.7
3,000	6 (7)	5 (2.2)	19 (7.8)	6 (3.9)	6 (0.8)	31 (4.8)	99.3	96.4	93.5
4,000		7 (3.1)	2 (0.8)		8 (1.1)	2 (0.3)		97.5	93.8
5,000		1 (0.4)	1 (0.4)		1 (0.1)	1 (0.2)		97.7	94.0

#### Procedure

The first group of participants took all the tests. All the tests were taken during normal class time. It took 2 weeks to complete the tests.

The study was administered in three sessions of 30 min each. Three listening comprehension measures were delivered in the first session. In order to balance the order effects, the three listening tests were completed in a Latin Square design. Then, after a 10-min break, the participants were required to take the Vocabulary Levels Test in the second session. Several days later, the participants were given the Productive Vocabulary Levels Test in the third session.

The second group of participants took the Vocabulary Levels Test and the Productive Vocabulary Levels Test.

### Data Analysis

The obtained data were analyzed with SPSS version 24. First, correlational analyses were performed to determine the relationship between the two types of vocabulary knowledge and the three English listening comprehension measures. Second, three pairs of hierarchical multiple regression analyses were conducted to address the research questions that concern the relative contribution of VB and VD across different listening comprehension measures—passage dictation, conversation, and passage (scores on VD and VB as independent variables, and scores on passage dictation, conversation, and passage as dependent variables). The control variables (age and years of learning English) were entered in step 1. VB and VD were entered in steps 2 and 3, and in the opposite order in steps 2A and 3A, to determine their unique contributions. Before multiple linear regression analysis, the data were checked for normality assumptions by the Kolmogorov–Smirnov test. All the data met normality assumptions. In addition, the data were checked for linearity, multicollinearity, and homoscedasticity. All the data met these three assumptions.

## Results

### Descriptive Statistics

The maximum and minimum scores, means, standard deviations, and reliability coefficients of all the measures are shown in Table 5. The mean scores in passage dictation, conversation, and passage tests suggest that the three tests were not demanding for the participants. The mean scores in VB and VD suggest that the VD measure was more difficult than that of VB for the participants.

**Table 5:** Descriptive statistics for all measures.

**All measures**	**Maximum**	**Minimum**	**Mean**	**SD**	**Reliability**
Dictation	14	2	9.519	2.422	0.950^a^
Conversation	10	4	7.584	1.462	0.616^b^
Passage	10	2	7.460	1.439	0.599^b^
VB	135	3	94.620	17.434	0.918^b^
VD	55	9	31.520	9.420	0.819^b^
Age	23	19	20.510	0.937	
YEL	16	6	10.340	1.958	

*VD, vocabulary depth; VB, vocabulary breadth; YEL, years of English learning. N = 113*.

a *Indicates inter-rater reliabilities*.

b *Indicates Cronbach's alpha reliabilities*.

It is worth noting that the two multiple choice tests displayed a relatively low reliability coefficient. The reason for this low alpha coefficient might be that the participants in this study were very homogenous and did not produce much variance in the two listening tests (SD = 1.462; SD = 1.439), which could lead to deflation in reliability estimate (Davies et al., 1999).

### Research Question One: What Is the Relationship Between Vocabulary Depth and Vocabulary Breadth?

As presented in Table 6, VB and VD had a different correlational relationship with the three listening task types. VD produced a moderate correlation with passage dictation (*r* = 0.581), but it produced a weaker correlation with conversation comprehension (*r* = 0.248) and passage comprehension (*r* = 0.317). Similarly, VB produced a moderate correlation with passage dictation (*r* = 0.429), but it produced a weaker correlation with conversation comprehension (*r* = 0.241) and passage comprehension (*r* = 0.295). Additionally, the correlation between VB and VD reached 0.543, which indicated that these two kinds of vocabulary knowledge were overlapped and interconnected constructs. Finally, the years of learning English of the participants had almost no correlation with other relevant dependent variables, indicating that there were no associations between the years of learning English of the participants and their vocabulary and listening comprehension performance.

**Table 6:** Correlations between both vocabulary knowledge and three listening measures and years of English learning.

	**Dictation**	**Dialogue**	**Passage**	**VD**	**VB**	**YEL**
Dictation	1					
Dialogue	0.34^**^	1				
Passage	0.322^**^	0.404^**^	1			
**VD**	0.581^**^	0.248^**^	0.317^**^	1		
**VB**	0.429^**^	0.241^**^	0.295^**^	0.543^**^	1	
YEL	0.148	0.09	0.04	0.102	0.065	1

*VD, vocabulary depth; VB, vocabulary breadth; YEL, years of English learning. N = 113*.

** *P < 0.01*.

### Research Question Two: Does the Relative Contribution of VD and VB to Listening Comprehension Depend on How Comprehension Is Assessed?

To probe the answer for the second question, a series of hierarchical regression analyses was conducted. The results are displayed in Table 7.

**Table 7:** Hierarchical regressions predicting passage dictation, conversation, and passage.

**Predictors**	**Dictation Δ*R*^**2**^**	**Conversation Δ*R*^**2**^**	**Passage Δ*R*^**2**^**
Step 1. VB	0.173^***^	0.071^**^	0.057^*^
Step 2. VD	0.159^***^	**0.023**	0.041^*^
Step 1A. VD	0.301^***^	0.068^**^	0.085^**^
Step 2A. VB	0.031^*^	**0.026**	**0.013**

*N = 113*.

* *p < 0.05*;

** *p < 0.01*;

*** *p < 0.001*.

The important results for the study were in steps 2 and 2A: VB and VD each predicted passage dictation significantly with VD being much stronger, whereas none of VB and VD could play a separate role in conversation comprehension. In addition, VD (after controlling VB) could predict passage comprehension task significantly while VB (after controlling VD) could not play a separate role in passage comprehension task.

## Discussion

This study investigated the relative effects of two dimensions of vocabulary knowledge on three different listening comprehension measures. The results showed that the relative contribution of VD and VB to listening comprehension depended on how listening comprehension was assessed.

### The Relationship Between Vocabulary Breadth and Vocabulary Depth

In this study, the correlation between VB and VD was not particularly strong (*r* = 0.543, *p* < 0.01) for intermediate Chinese English learners. Previous studies that measured VD through the Word Association Test found correlations (*r* from 0.52 to 0.82) between VD and VB (Nurweni and Read, 1999; Qian, 1999, 2002; Greidanus et al., 2004; Zhang, 2012). Other studies that measured VD through the Productive Vocabulary Levels Test found correlations (*r* from 0.67 to 0.76) between VD and VB (Zhang, 2011; Wang, 2015). Compared with these studies, this one indicated a weaker correlation between VB and VD. Nurweni and Read (1999) and Akbarian (2010) found that the relationship between VB and VD is related to the language proficiency level of learners, as indicated by a higher relationship between the two dimensions for relatively advanced language learners and a lower relationship for less advanced language learners (Nurweni and Read, 1999; Akbarian, 2010). In the study of Zhang (2011), participants were from Beijing International Studies University (BISU) with foreign languages and literature as the dominant discipline. It can be inferred that the English proficiency of the participants in that study was higher than that of the participants in this study. In the study of Wang, although chosen randomly from three natural classes in a medical University, some participants especially poor in English were eliminated according to their academic performances in English exams. Additionally, all participants had prepared for College English Test Band 4 for several months. Consequently, the English proficiency of the participants in that study might be higher than that of the participants in this study. This may explain why this study revealed a weaker correlation between VB and VD. Further studies with different measures for VD are needed to test the relationship between VB and VD.

### The Relative Contribution of Vocabulary Depth and Vocabulary Breadth to Second Language Listening Comprehension

The relative contribution of VD and VB to listening comprehension depended on how listening comprehension was assessed. Specifically, both VD and VB significantly predicted passage dictation performance after controlling each other. However, VD was a major contributor to the passage dictation measure. Dictation is an integrative test to assess listening, decoding, and spelling, etc., and a synthesis of the speech perception process at the phonological, syntactic, and semantic levels (Flowerdew and Miller, 2005) to test more than simple word recognition and spelling (Oakeshatt-Taylor, 1977). On one hand, passage dictation requires general understanding of a text. Knowing more words undoubtedly helps to get the main idea of a passage. It is no doubt that VB plays a significant role in dictation to assess general understanding. On the other, passage dictation requires participants to write down the correct form of every word they have heard. The VD measurement in this study also requires the participants to provide the correct spelling of words. It is reasonable that understanding more about words is helpful in extracting meaning from the text and in constructing meaning and form of the text.

Noticeably, neither VD nor VB could separately predict conversation comprehension performance after controlling each other. On one hand, the conversations were overall lexically simple, and the words may have been largely known to all the participants. Consequently, VB could not play an independent role in conversation comprehension. On the other hand, the comprehension questions for the conversations tested mainly the literal understanding of participants, which did not require deeper processing of words and their meaning relationships. As a result, VD could not play a separate role in conversation comprehension either.

Importantly, VD (after controlling VB) significantly predicted passage comprehension performance while VB (after controlling VD) could not. Although with the same multiple choice format as conversations, the questions mainly focused on reorganizing information or making inferences, which required the participants to process the words deeply with deeper cognitive demand to construct a situation model. More specifically, much more knowledge of words in the passages would be needed for successful textual inferential understanding and construction of a situation model (Kintsch, 1998). In addition, with the expository text genre, the passages were much more formal than conversations, and the topics of passage comprehension are a bit more difficult than those of conversations. As a result, it makes sense that knowing words well would help to construct meaning and make inferences in complicated passage comprehension. The assessment focus of passage comprehension measure here indicated a similarity in cognitive demands to the short passage comprehension task in Zhang and Yang (2016), where Chinese learners' VD was found to be a stronger predictor than VB.

A potential problem is that for both conversation and passage comprehension with multiple choice format questions, participants might use pragmatic test-taking strategies, such as searching for keywords in the answer options and guessing, to avoid creating a situation model or even much of a macro-structural hierarchy of propositions as described by Kintsch (1998). In the future, when choosing listening comprehension tasks, researchers should pay attention to what skills they are measuring, because different listening comprehension tasks measure and depend upon different skills. This needs to be confirmed by further studies with different participants.

In this study, there were no associations between the years of learning English of participants and their vocabulary and listening comprehension performance. Theoretically, the longer participants have learned English, the more time and opportunities they have had to access English. As a result, they might have increased VB and VD, and English listening proficiency. However, the years of learning English of the participants do not guarantee the same degree of active English learning or opportunities for English use across all participants. Therefore, the intensity and the extent of English input (the amount of experiences and opportunities for using English) need to be considered in future studies.

In sum, this study suggests that the relative contribution of VD and VB to L2 listening comprehension varies across different listening comprehension measures. These results are consistent with those found in the relationship between two dimensions of vocabulary knowledge and reading comprehension (Li and Kirby, 2015; Zhang and Yang, 2016). In addition, this study tentatively supports the idea that VD has a stronger relationship with various listening measures (especially with passage dictation and passage comprehension). These results are in agreement with those of some studies, which support the stronger effects of VD on listening comprehension (Teng, 2014; Wang, 2015; Dabbagh, 2016; Farvardin and Valipouri, 2017). However, the results are inconsistent with those of some studies that conclude that VB is the basic component of vocabulary knowledge in listening comprehension and that VD contributes very little to successful listening comprehension (Stæhr, 2009; Wen, 2014; Migdadi et al., 2019). The contradictory results might be attributed to different listening texts and task types used in these studies, that is to say VD and VB might have relative effects on different kinds of L2 listening texts and tasks.

### Implications

Pedagogically, the findings from this study have some implications for second language teaching and assessment. The findings here attract our attention to the need for enhancing the vocabulary of L2 learners in a classroom to enable them to have a better performance in L2 listening. Activities promoting VD that emphasizes the form-recall knowledge are highly recommended. In addition, this study also can offer suggestions for L2 listening comprehension test designers. The findings reveal that listening comprehension measures should include a variety of text types that address varied listening skills, which can tap both VD and VB. In this way, a positive washback effect would be produced on vocabulary teaching and learning.

Theoretically, the findings are consistent with those of two studies that explored the relative effects of VB and VD on different L2 reading measures (Li and Kirby, 2015; Zhang and Yang, 2016). The results call for a special concern that some commonly used listening comprehension measures may tap different cognitive processes. Both individual differences in listener characteristics, such as vocabulary, and characteristics of the listening measure, such as skills measured, influence L2 listening performance (Wallace and Lee, 2020). As a result, the relative effects of VB and VD on different listening measures may be influenced in different degrees by particular skills that can have some effects on comprehension. Therefore, inconsistent conclusions across previous studies on the relative contribution of VB and VD to L2 listening comprehension may be attributed to different listening measures used in these studies. Future studies can re-examine and disentangle the effects of variation with more refined listening comprehension measures.

### Limitations and Future Research

This study has some limitations. First, it is worth noting that the Vocabulary Levels Test assesses knowledge of the written form of a word, whereas listening involves recognizing the spoken form of a word. This might constitute a potential problem that a word recognized in its written form will not necessarily be recognized in its spoken form. Moreover, orthographic word knowledge is undoubtedly a prerequisite for the ability to read and write but is less important for listening and speaking, whereas phonological word knowledge is highly important for listening and speaking but is less important for reading and writing. Indeed, future studies to investigate the effects of the two types of vocabulary knowledge on English listening should be based on a vocabulary test that involves hearing the target words rather than reading them. Second, in this study, only one depth measure was used to measure VD. Given the complex nature of VD, there is a need to employ different measures of VD, such as the Word Associates Test of Read, based on the comprehensive conceptualization of this construct. Third, in this study, the VD measure assessed productive orthographic knowledge, while the passage dictation test that required the participants to spell words correctly tapped into productive phonological knowledge. Future studies should choose the listening test carefully to avoid this kind of problem.

## Conclusion

This study investigates the relative contribution of VB and VD to three different listening comprehension measures. The results showed that the relative contribution of VD and VB to the listening comprehension of Chinese English learners varied across listening comprehension measures. The findings suggest that listening comprehension measure can influence the relationship between vocabulary knowledge and L2 listening comprehension. This may be because text type and question type affect listening comprehension. As a result, they influence the interaction between vocabulary knowledge and L2 listening comprehension.

## Data Availability Statement

The original contributions presented in the study are included in the article/supplementary material, further inquiries can be directed to the corresponding author/s.

## Ethics Statement

The studies involving human participants were reviewed and approved by Anhui Agricultural University. The patients/participants provided their written informed consent to participate in this study.

## Author Contributions

YL wrote the manuscript. HS and LW revised the manuscript. XZ designed the study. All authors contributed to the article and approved the submitted version.

## Conflict of Interest

The authors declare that the research was conducted in the absence of any commercial or financial relationships that could be construed as a potential conflict of interest.
